# Dysfunctional self-talk associated with eating disorder severity and symptomatology

**DOI:** 10.1186/2050-2974-2-14

**Published:** 2014-05-27

**Authors:** Ned Scott, Tanya L Hanstock, Chris Thornton

**Affiliations:** 1 Clinical Psychology Program, School of Behavioural, Cognitive and Social Sciences, University of New England, Armidale, NSW 2351, Australia; 2 School of Psychology, University of Newcastle, Callaghan, NSW 2308, Australia; 3 The Redleaf Practice, Wahroonga, NSW 2076, Australia

**Keywords:** Self-talk, ED severity, ED symptomatology

## Abstract

**Background:**

While self-talk has been argued to play a crucial role in the development and maintenance of eating disorders (EDs), it has received limited research attention. This study aimed to explore the relationship of ED self-talk with ED severity and symptomatology.

**Methods:**

Analysis of the existing literature, supplemented with a small-scale pilot study, identified 24 distinct categories of ED self-talk. The main study involved the completion of on-line questionnaires by 172 women aged 18–49, recruited through clinical services, ED websites, and the general population. Participants were assigned to clinical (*n* = 83) and non-clinical (*n* = 89) samples, using the Eating Disorder Examination Questionnaire to screen for ED psychopathology.

**Results:**

Substantial differences in the levels of ED self-talk were found between the clinical and non-clinical populations. Principal components analysis, conducted within the clinical sample, revealed ED self-talk to have a two-component structure. Self-talk reflecting an ‘abusive relationship’ between the sufferer and the ED strongly predicted overall severity and several aspects of symptomatology. ‘Ascetic attitudes’ towards thinness were linked with compulsive exercising and lower BMIs but not with overall severity.

**Conclusions:**

Close examination of the ‘abusive relationship’ component suggests a need to loosen the connection between negative appraisals of the abused self and the abusive voice of the ED so that the former can fulfil their potential as a force for change. Further, in seeking to counter the impact of the ED voice, it is suggested that the seducer and abuser roles require primary clinical focus.

## Background

Eating disorder (ED) self-talk or the ‘voice of A/b (anorexia/bulimia)’ ([1], p. 21) has been argued to play a crucial role in the development and maintenance of disordered eating behaviour. This voice ensures that weight, shape, and eating issues are never far from one’s consciousness [2], that self-worth remains integrally connected with thinness [3], and that the sufferer’s original self-identity and values are subjugated, along with thoughts of a healthier/more positive nature [1,4].

Insight into the nature of ED self-talk comes primarily from anecdotal self-reports obtained during the course of clinical treatment e.g., [1] and from first-hand accounts written by past sufferers e.g., [5]. In addition, several exploratory studies have made initial attempts at categorising ED self-talk or cognitions e.g., [2,4,6-8].

An examination of this material reveals the multi-dimensional nature of ED self-talk. ED sufferers often refer to an ED voice, which, in some instances, speaks to them in the second person and which they perceive as having a different persona, despite it being essentially ego-syntonic and non-psychotic in nature [4]. For example, De Rossi ([5], p. 238) refers to her ED voice as the “drill sergeant”. Maisel et al. [1] provide examples of four different roles adopted by the ED voice: the seducer, making promises to remove pain and suffering; the coach, monitoring eating behaviour and providing guidance and exhortation; the mentor or “voice of reason” (p. 22) that helps the sufferer interpret the world through eyes that see thinness and self-discipline/denial as core moral virtues; and the abuser/bully, seizing every opportunity to denigrate the sufferer as unworthy. Tierney and Fox [9] analysing first hand reports by individuals with anorexia nervosa (AN), suggest that the sufferer’s relationship with the voice changes markedly over the course of the ED, as the seducer role becomes less prominent and the abuser role more so.

The literature also details a form of self-talk that represents sufferers’ appraisals of the ED’s impact on their lives. These appraisals reflect the ambivalence felt by many sufferers towards their ED [8]. Hence, on the one hand, there is self-talk that reflects the sufferer’s identification with the ED, their pride in what they have achieved as a result, and their sense that their ED is essential to their coping abilities. However, there is also self-talk that reflects the suffering inherent in living with an ED, the weariness resulting from constantly worrying about food, and the sense of loss regarding identity and life ambitions [7].

In this study, we define ED self-talk as including both these aspects: the so-called voice of the ED and the voice of the individual herself. We include all cognitions/inner verbalisations made by the individual that relate to self-worth, eating behaviours, or weight/appearance.

Sufferers’ first-hand accounts document the distress they experience as a result of ED self-talk, and also clearly outline the influence they believe the ED voice has in maintaining the ED. Despite this, these accounts also testify to the fear associated with the idea of being separated from that voice e.g., [1,5,9]. Tierney and Fox [9] have linked the power inherent in the ED voice with the high rate of relapse in AN. Maisel et al. [1] argue that treatment efforts have to centre on lessening the hold the “voice of A/b” has on the sufferer. Higbed and Fox [4] suggest that better understanding ED sufferers’ beliefs about this voice may be central to reducing its power. Williams and Reid [8] argue that the failure of some health professionals to acknowledge the hold of such pro-cognitions is an important factor in increasing treatment-resistance. Despite this, little attention has been paid to systematically documenting such cognitive styles [2], nor to measuring their change as a result of treatment [10].

### Aims

The first objective of this study was to produce a detailed categorisation of ED self-talk so as to better inform clinicians’ dealings with such patients. Additionally, the study aimed to investigate the factor structure of such self-talk and the extent to which the factors identified were associated with overall severity and specific forms of ED pathology (e.g., successful dietary restriction versus purging). Specifically, it was hypothesised that:

1. Women with EDs would experience self-talk related to eating, weight, and self-worth that, in its nature and frequency, was qualitatively and quantitatively distinct from that experienced by their same aged peers in the general population.

2. Within the clinical sample, factor components of this self-talk would predict both overall severity and the strength of specific forms of ED pathology: namely, restrictive eating, binge eating, purging, compulsive exercising and body mass index (BMI).

## Method

### Exclusion criteria

Males were excluded since the vast majority of patients presenting with EDs are female [11]. Additionally, the survey was limited to adult women aged under 50. Females under 18 years of age were excluded, partly due to ethical safety concerns, and also because of the potential additional impact of lifestage on the nature of their self-talk. For the latter reason, women aged 50 and over were also excluded.

### Initial pilot study

**Figure 1:**
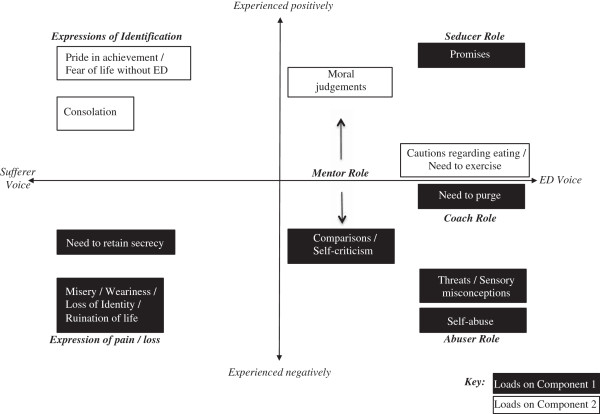
Segmentation of eating disorder (ED) self-talk components based on existing literature.

Analysis of the existing literature produced an initial list of 21 distinct categories of ED self-talk. Based on this review, these different forms of ED self-talk identified were plotted against two dimensions: the ED voice versus the sufferer’s voice and whether talk is experienced positively or negatively (see Figure 1).

A small-scale pilot study was conducted to confirm/refine this list of categories and develop first person sample items to represent each category. Participants in the pilot were six women with a history of eating disorders, five of whom had been diagnosed with the restricting form of AN, and one with the purging type of Bulimia Nervosa (BN). At the time of participation, half had current diagnoses of Eating Disorder Not Otherwise Specified (EDNOS) and half were classed as being in remission. Participants were recruited by providing information on the study to clinicians and sufferers, who were encouraged to pass on the material to clients/fellow sufferers. Interested parties then contacted the main researcher (NS) by email. Individuals who were personally known to the researchers were excluded from participation. Methods used to explore self-talk included the keeping of a two-day diary, telephone qualitative interviews, and semi-structured email questionnaires.

Qualitative content analysis [12] was utilised to analyse these pilot interviews. The pilot study served to confirm the presence of all 21 self-talk categories identified in the literary analysis, and also uncovered a further three categories, which were subsequently included in the main study. Content analysis was used to formulate sample first person statements to represent each category as detailed in Table 1.

**Table 1:** Categories of ED self-talk identified

**Description**	**Example selected for inclusion**
**Promises** (of reduced pain)	*If I’m thinner/lose more weight then rejection and criticism won’t hurt so much.*
**Consolation**	*I may not be doing so well in some areas of my life but at least I’m thin.*
**Self-congratulations** (on eating behaviour etc.)	*I’m doing really well in controlling my eating today.*
**Threats**	*If I don’t keep my eating under control, I’m going to get obese and be rejected by everyone.*
**Cautions regarding eating**	*That food/eating situation is dangerous. I’m going to have to be really careful about what I eat.*
**Need to purge**	*I’m worried that I’ve eaten too much. I need to get rid of it by purging.*
**Need to exercise**	*I can’t afford to just sit around, I have to exercise more.*
**Self-disappointment** (at ability to meet dietary ideals)	*I’m so disappointed at myself: I’ve failed to live up to my dietary ideals again.*
**Self-abuse**	*I am so pathetic and useless. I have no discipline or self-control.*
**Self-punishment**	*Because I have failed to live up to my dietary ideals I deserve to be punished by denying myself food or exercising harder.*
**Self-criticism** (lack of self-worth)	*I’m such a failure. I try really hard to do well and make friends but I’m just not the type of person I want to be.*
**Comparisons** (with others)	*Look at her she’s so thin and she does it so easily. Why can’t I be like her?*
**Reinterpretations**	*When they say things like “you’re looking well”, what they’re really meaning is how much weight I’ve put on.*
**Sensory misperceptions**	*Just feel that stomach/Look at those thighs (etc.). I feel/look so fat/terrible.*
**Denial** (of symptoms)	*Feeling tired and/or cold doesn’t mean there’s anything wrong with me. I just have to be stronger.*
**Moral judgements**	*Practising self-restraint is an important virtue. I despise people who have no self-control.*
**Ruination of life**	*My concern about food and weight is threatening the other things I want to achieve/get out of life.*
**Weariness** (of constantly thinking about food)	*I’m so fed up of constantly thinking about food and what I eat.*
**Misery** (of living with an ED)	*Life is so miserable/lonely living like this.*
**Lost identity**	*I feel like I don’t really know who I am/what I stand for any more. I’ve lost touch with the real me.*
**Need for secrecy**	*People mustn’t find out the way I think or behave concerning food.*
**Rebellion**	*I’m fed up of trying to control what I eat. I’m just going to eat whatever and as much as I want!*
**Pride in ED**	*When I think about how self-controlled I am around food compared to the average person, I feel quite proud of what I can achieve.*
**Fear of life without ED**	*If I let go of my strict control and ideals around eating, I’m really quite frightened about what life would be like.*

### Main study

Participants included 172 women aged 18 to 49 (*M* = 28.86, *SD* = 9.32). Recruitment involved the use of posters/flyers advertising the study. Two versions were produced, one aimed at women with EDs and the other directed to all women in the study’s age range. The former poster/flyer was placed, with clinicians’ permission, in a number of private ED in-patient clinics, handed directly to outpatients by treating private clinicians and dietitians, and also placed on a number of ED websites. The latter version was distributed through friends and colleagues. Details of the study were also provided to University of New England (UNE) students enrolled in first year psychology units. Students who chose to participate were awarded one Research Participation (RPO) point upon completion. No other incentives were provided for study participation.

### Research design

The study employed a cross-sectional case-control design, using the Eating Disorder Examination Questionnaire EDE-Q [13] to screen for the presence of ED pathology. Screening criteria were based on those recommended by Mond, Hay, Rodgers, Owen, and Beumont [14] who suggest that a positive screen should require both a high score (2.3 or more) on the EDE-Q global scale and the confirmatory presence of some behavioural symptoms. Using the presence of some objective bulimic episodes (OBEs) and/or compulsive exercising for weight/shape reasons at least weekly as confirmatory symptoms, Mond et al. achieved a sensitivity of 0.83, a specificity of 0.96, and a positive predictive value of 0.56. In this study, the presence of some form of purging was also considered as a confirmatory behavioural symptom. On the basis of these amended criteria, 78 participants were assigned to the clinical sample.

Additionally, participants whose reported physical/behavioural symptoms were sufficient to clinically justify an EDNOS diagnosis in their own right were also included in the clinical sample, irrespective of their EDE-Q scale score. Accepted criteria in this respect were the bi-weekly presence of OBEs and/or purging, and/or a BMI of less than 17.5, the accepted criteria for AN [15]. A further 5 participants were added to the clinical sample as a result. In total, 83 women were assigned to the clinical sample (*M* [age] = 27.01, *SD* = 8.32) and the remainder to the non-clinical sample (*M* = 30.58, *SD* = 9.89). An independent samples *t* test revealed that this difference in age was significant, *t* (168.2) = 2.57, *p* = .011, *d* = 0.39.

### Procedure

Ethical approval for the study was granted by UNE’s Human Research Ethics Committee. Questionnaires were administered online using Qualtrics. Prospective participants were provided with codes to access the study. Initial screens explained the nature of the research, prior to seeking participants’ informed consent and checking their eligibility.

Questioning in the main study asked participants to first identify which of the 24 categories of self-talk they themselves experienced. For each type mentioned, participants were asked to specify how often they experienced that type of self-talk on an 8-point Likert scale, ranging from 1 (*no longer experienced*) to 8 (*every waking hour*). This was expanded to a 9-point scale by giving those participants who did not specify an item a score of zero.

### Additional measures

#### Severity/Nature of ED pathology

The current level and nature of core ED pathology was assessed using the EDE-Q. The EDE-Q contains three attitudinal subscales covering Eating Concern, Shape Concern, and Weight Concern. A further subscale, entitled Restraint, measures the frequency of attempting to restrict the amount and types of food eaten. All items relate to the preceding 28-day period. The global scale is defined as the mean of these four subscales and is considered to give an overall measure of ED pathology. EDE-Q subscales have been shown to have good internal consistency amongst both community samples of women [16] and women with bulimic symptoms [17]. Cronbach’s α*’*s for the current study ranged from .86 for the Restraint subscale to .94 for Shape Concern.

#### Impact on psychosocial functioning

The overall impact of the ED on psychosocial functioning was assessed using the Clinical Impairment Assessment Questionnaire CIA [18]. The CIA asks the participant to specify to what extent, over the last 28 days, their eating habits, exercising, and feelings about eating, shape, or weight have impacted on particular areas of their life. The CIA has high internal consistency (α = .97), acceptable test-retest reliability (*r* = .86), good construct and discriminant validity, and has been demonstrated to be sensitive to change [19]. Cronbach’s *α* for the current study was .98. Fairburn [20] argues that impact on psychosocial functioning is a further valuable indicator of the ED’s hold on the individual.

#### Current and past diagnoses and treatments

Participants were also asked to supply details of any current and past ED diagnoses, perceived periods of remission, and the forms of community or inpatient treatment they had received.

## Results

### Data cleaning

Of the 216 individuals who consented to participate in the study, 42 dropped out prior to completing the EDE-Q and thus could not be assigned to either the clinical or non-clinical samples. Examination of a further two response sets (revealed by Mahalanobis distance calculations to be extreme multivariate outliers within the clinical sample) suggested these cases were not valid members of the clinical population. Hence they were excluded, leaving a total of 172 participants.

The data set was complete for these participants with the exception of two who did not complete the CIA scale and a further seven who omitted/gave nonsensical values for their weight and/or height, and whose BMI was therefore incalculable. Visual examination of other key variables for these participants revealed no systematic pattern of response; hence in analyses of the variables concerned, cases with missing values were simply excluded.

### Outliers and violated assumptions

Independent samples *t* test comparisons of self-talk and ED severity measures between the clinical and non-clinical samples were complicated by the lack of normality for many of these measures, particularly in the non-clinical sample. Where possible, data was transformed to address this. A transformation was also required when a paired sample *t* test was used to compare mean frequency scores in the clinical and non-clinical samples across the 24 self-talk items. In all instances, the impact of these transformations on results was inconsequential, so the untransformed data is reported to enhance ease of interpretation. Where the data was too severely non-normal to permit transformation, the non-parametric Mann–Whitney test was substituted. For the independent samples *t* test used to compare the number of self-talk items experienced, Levene’s test revealed the assumption of homogeneity of variance was violated. Hence, the results reported are those for equal variances not assumed. In contrast, few problems were encountered with the assumptions relating to the multiple regression analyses conducted. The data was analysed using IBM SPSS Statistics Version 18.

### Participants included in the study

Thirty-six (21%) of the 172 participants reported having a current ED diagnosis, all but two of which (both of whose current diagnosis was AN in remission) satisfied the EDE-Q criteria for inclusion in the clinical sample. The current diagnoses reported were AN by 12 (7%) participants, AN in remission by 11 (6%), BN by 4 (2%), and EDNOS by 9 (5%). A further 20 (12%) participants reported past diagnoses. In total, 35 (20%) of participants had had a diagnosis of AN at some point, 20 (12%) a diagnosis of BN, and 17 (10%) a diagnosis of EDNOS.

In total, 83 (48%) participants satisfied the criteria for the clinical sample. Within this clinical sample, 42 (51%) participants had had a formal diagnosis at some time; 59 (71%) reported OBE’s and 33 (40%) reported some form of purging in the last 28 days. Weekly episodes of compulsive exercising for weight/shape reasons were reported by 41 (49%). Mean scores for both samples on scales measuring ED severity and clinical impairment are detailed in Table 2. To ensure differences between the clinical and non-clinical samples held across all these scales, a series of Mann–Whitney tests were conducted. All were significant with *p* < .001. Effect sizes range from *d* = 1.90 to 2.93 (Table 2).

**Table 2:** Mean scores (S.D.s) on severity measures across clinical and non-clinical samples

**Scale**	**Clinical sample**	**Non-clinical sample**	**Effect-size *d***
EDE-Q			
Global scale	4.09 (1.06)	1.22 (0.89)	2.93***
Restraint subscale	3.56 (1.54)	1.05 (1.06)	1.90***
Eating concern subscale	3.29 (1.54)	0.58 (0.85)	2.20***
Shape concern subscale	4.96 (0.97)	1.79 (1.25)	2.82***
Weight concern subscale	4.53 (1.10)	1.43 (1.18)	2.71***
CIA	28.07 (12.52)	4.80 (6.51)	2.36***

Note: EDE-Q = Eating Disorder Examination Questionnaire; CIA = Clinical Impact Assessment Questionnaire; ***p < .001.

### Eating disorder self-talk

On average, participants in the clinical sample identified with 12.02 (50%, *SD* = 5.39) of the 24 categories of self-talk presented. This compared with an average of 4.38 (18%, *SD* = 3.51) self-talk categories in the non-clinical sample. An independent samples *t* test confirmed this difference was significant, *t* (139.4) = 10.93, *p* < .001, *d* = 1.68. Multiple regression analysis confirmed that membership of the clinical sample remained a highly significant predictor, *p* < .001, when age was controlled for.

All categories of self-talk were experienced to some degree across both samples. However, as shown in Table 3, in the clinical sample, a substantial proportion experienced each type of self-talk at least several times per day. In the non-clinical sample, such frequencies were extremely rare.

**Table 3:** Percentages of sample experiencing different types of eating disorder (ED) self-talk

	**Clinical sample**		**Non-clinical sample**	
**Self-talk category**	**At least several times per day %**	**Ever experience %**	**At least several times per day %**	**Ever experience %**
Sensory misperceptions	59	93	2	54
Comparisons	33	86	4	53
Threats	30	64	-	11
Need for secrecy	29	53	-	11
Need to exercise	28	55	8	42
Self-disappointment	25	70	2	25
Self-abuse	25	69	2	19
Ruination of life	25	54	-	11
Lost identity	25	52	-	8
Self-criticism	25	41	1	16
Fear of life without ED	22	45	1	8
Self-punishment	22	40	-	7
Weariness	22	55	-	12
Need to purge	19	45	-	9
Misery	19	45	-	7
Cautions re. eating	18	55	1	17
Reinterpretations	14	47	-	7
Promises	12	34	1	13
Consolation	10	35	-	15
Self-congratulations	8	63	-	42
Moral judgements	7	24	1	12
Denial	6	35	-	7
Pride in ED	6	23	1	10
Rebellion	4	35	-	21

Additionally, a paired sample *t* test was used to compare the mean frequencies with which each of the 24 self-talk items was experienced in the two samples. As measured on the 9-point Likert scale, mean frequency scores in the clinical sample (*M* = 2.53, *SD* = 1.07) were significantly greater than in the non-clinical sample (M = 0.57, *SD* = 0.50), *t *(23) = 11.60, *p* < .001, *d* = 2.50.

### Principle components analysis

To explore the factor structure of ED self-talk, the data collected from all clinical sample participants on these 24 items was subjected to Principle components analysis (PCA). Prior to the final analysis, four items (*Self-congratulations*, *Self-disappointment*, *Denial*, and *Rebellion*) were omitted due to low communalities (less than .2), and a further two items (*Self-punishment* and *Reinterpretations*) omitted due to cross-loadings. The Kaiser-Myer-Olkin measure of sampling accuracy was .81, indicating strong linear relationships within the item set. Communalities for the retained items were all above .25, indicating that they all contributed a meaningful amount of shared variance to the data set.

Both Cattell’s [21] scree test and Velicer’s [22] minimum average partial (MAP) test indicated that two components should be retained. Direct oblimin rotation was used with Δ set to zero to permit correlations between components. Table 4 shows the component loadings after rotation. Component 1 has over four loadings in excess of .6, indicating it is reliable despite the relatively small sample size [23]. Component 2 has only three loadings in excess of .6 and therefore findings in relation to this factor need to be treated with a degree of caution.

**Table 4:** Rotated component loadings and Cronbach’s α for eating disorder (ED) self-talk item frequencies

	**Component 1**	**Component 2**
	**Abusive Relationship**	**Ascetic Attitudes**
**Variable**	**(*α*** **= .88)**	**(*α*** **= .72)**
Threats	.79	
Comparisons	.71	
Self-criticism	.71	
Self-abuse	.69	
Sensory misconceptions	.66	
Need to purge	.65	
Misery	.62	
Lost identity	.55	
Ruination of life	.54	
Weariness	.54	
Promises of reduced pain	.51	
Need to retain secrecy	.51	
Moral judgements		.75
Pride in ED		.73
Fear of life without ED		.60
Consolation		.55
Cautions re. eating		.49
Need to exercise		.44

Note: Rotation employed the direct oblimin approach with Δ = 0. Loadings below .4 are not included. As recommended by Tabachnick and Fidell [24] pattern matrix loadings are reported. These values are partial scale correlations between the scale items and the components after controlling for the variance shared by the other retained components.

Examination of these loadings suggests that component 1 combines both sides of the abusive relationship that characterises much of ED self-talk: self-denigration and self-pity; whereas component 2 is connected with a moral or ascetic attitude to thinness. Together, these components accounted for 45% of the variance in these self-talk items. Individually, component 1 accounted for 31%, component 2 for 19%. The correlation between the two components was .27, indicating a moderate relationship between the two factors.

Scale scores were computed by averaging the items that loaded above .4 on each component. Cronbach’s α for both scales exceeded .7 reflecting adequate internal consistency. Because scale scores were heavily skewed (particularly in the non-clinical sample), the Mann–Whitney *U* test was employed to evaluate differences between scale scores for the clinical (*n* = 83) and non-clinical (*n* = 89) samples. For both self-talk components, scores were significantly greater in the clinical sample. For Abusive Relationship, the clinical sample had a mean score of 3.04 (*SD* = 1.85) compared to 0.58 (*SD* = 0.61) for the non-clinical; *U* = 483.5, *p* < .001, *d* = 1.81. For Ascetic Attitudes, the clinical sample had a mean score of 1.92 (*SD* = 1.65) compared to 0.58 (*SD* = 0.77) for the non-clinical; *U* = 1640, *p* < .001, *d* = 1.05.

### Relationship between components and eating disorder severity

The two measures of severity, the global EDE-Q and the CIA were found to be highly correlated in the clinical sample, *r* (81) = .81, *p* < .001. Accordingly, a measure of overall severity was produced by averaging the standardised scores on these two variables. A multiple regression analysis was then conducted to determine, within the clinical sample, the extent to which ED self-talk predicts ED severity. The two self-talk components identified served as predictors, and overall severity served as the dependent variable. Table 5 presents the results of this analysis. Overall, the two self-talk components predicted 60% of the variance in ED severity. However, only the Abusive Relationship component was a significant individual predictor, accounting for 46% of the variance.

**Table 5:** Predicting eating disorder severity and symptomatology from self-talk components

	**Total variance explained %**	**Variance explained by individual factors**	
**Variable**		**Abusive Relationship %**	**Ascetic Attitudes %**
Overall severity	60***	46***	< 1
Specific symptoms:			
Restraint	46***	23***	< 1
Purging	17**	13**	2
OBE	9	7	6
Compulsive exercising	21***	< 1	12**
BMI^†^	24***	< 1	16***

Note: OBE = Objective Bulimic Episodes, BMI = Body Mass Index, ***Indicates p < .001, **Indicates p < .01. Analyses conducted on clinical sample only. *n* = 83, except for analysis marked † for which *n* = 80.

### Relationship between components and behavioural/outcome symptoms

Further regression analyses were conducted to assess the extent to which self-talk predicts specific ED symptomatology. Again the two self-talk components served as predictors. Dependent variables were OBE, Purging, Compulsive Exercising, Restricted Eating, and BMI. The first three were measured by the number of reported occasions over the last 28 days. Restricted Eating was represented by the EDE-Q Restraint Subscale score. BMI’s were calculated from participants’ reported weights and heights.

To determine the *p* value correction necessary for these analyses, the effective number of independent variables (Veff) represented by these five variables was estimated using a factor analysis as described by Nyholt [25]. Veff was 4.63, meaning for α = .05, *p* < .011 was required for significance.

Results of all five analyses are detailed in Table 5. Together, the two self-talk components significantly predicted Restricted Eating, Purging, Compulsive Exercise, and BMI, accounting for 45%, 17%, 21%, and 24% of the variance in these measures respectively. Individually, the Abusive Relationship component was a significant predictor of Restricted Eating (accounting for 24% of the variance) and Purging (13%), whereas the Ascetic Attitudes component was a significant predictor of Compulsive Exercising (12% of variance) and BMI (17%). The result for the regression analysis predicting OBE was not significant when the *p* value correction was applied.

## Discussion

The study set out to document the nature of ED self-talk and its relationships with ED severity and symptoms. Comparisons were made between ED self-talk in clinical and non-clinical samples and the factor structure of such self-talk within the clinical sample was investigated. The ability of the factors that emerged to predict ED severity and symptomatology was explored, along with the effectiveness of strategies used to counter this self-talk.

### The distinctive nature of eating disorder self-talk

The first hypothesis, that women with EDs (as classified using the EDE-Q) would experience a level of self-talk regarding eating, weight, and self-worth that was qualitatively and quantitatively distinct from their same aged peers in the general population, was strongly supported. Although all self-talk items were found in both samples, women in the clinical sample experienced more items, at greater average frequencies, than those in the non-clinical sample. Further statistical confirmation of this difference was obtained by comparing mean scores on the two self-talk components that emerged from the PCA on item frequency. For each component, those items loading upon it occurred with greater average frequency in the clinical than in the non-clinical sample. Effect sizes for these analyses were all large (greater than 1.0). Visual examination of the data provided an important qualitative distinction. Those with EDs commonly reported experiencing certain items several times each day (or more often). Such frequencies were extremely rare in the non-clinical sample.

### Self-talk components

The two components of self-talk that emerged from the PCA, Abusive Relationship and Ascetic Attitudes can be reviewed in the light of the theoretical segmentation of self-talk based on the literature (Figure 1). Both components include items considered to represent both the ED voice and the voice of the sufferer. This helps explain the difficulty individuals with ED have in distinguishing their voice from that of the ED [1]. The main distinction between the two factors is whether items are experienced positively or negatively, with the key exception that *Promises* load on the Abusive Relationship component (see below for further discussion), which otherwise consists primarily of negatively experienced items. The three advice components; *Cautions about eating*, *Need to exercise*, and *Need to purge*; can be viewed as containing both positive and negative experiential aspects [8]. However, the fact that *Need to purge* loads on the predominantly negative Abusive Relationship component is consistent with the shame that commonly accompanies these actions [3].

### Predicting eating disorder severity and symptomatology

Hypothesis 2 concerning the ability of these self-talk components to predict ED severity and specific symptomatology was supported with the sole exception that self-talk was not found to predict the frequency of OBEs. However, the two individual components predicted quite distinct aspects. Greater average frequency scores on the Abusive Relationship component predicted greater frequency of purging, greater attempts to restrict eating (as measured on the EDE-Q restraint subscale), and increases in overall ED severity. In contrast, greater average frequency scores on the Ascetic Attitudes component predicted greater frequency of compulsive exercising and lower BMI’s. Scores on this second component did not predict overall severity.

These findings are in contrast to past research asking sufferers’ to evaluate the impact (positive and negative) of their eating disorder on their lives. Where such beliefs are concerned, it is the strength of positive rather than negative ED cognitions that predict resistance to treatment [8,26] and lesser motivation to change [27]. At an experiential level, however, it is the strength of predominantly negative self-talk that predicts ED severity. This finding lends support to the contention of Maisel et al. [1] that treatment efforts should focus on lessening the hold the ED voice exerts on sufferers. Although the Abusive Relationship component includes several items representing the sufferer’s voice, these recognitions of the negative consequences of the ED would appear to have little impact in reducing the grip of the ED, presumably because they are so strongly linked with the ED voice, in both its abuser and seducer roles. According to Walker’s [28] cycle of violence theory, these two roles mirror those adopted by perpetrators of domestic violence, which Walker argues explain the difficulty victims of such violence have in leaving those relationships. In the case of EDs, however, the ‘perpetrator’ is in the victim’s head.

The fact that the two self-talk components predict distinct behavioural and physical symptoms would, at first sight, seem to run counter to Fairburn’s [20] contention of a common ED psychopathology. However, comparing the relationships between Ascetic Attitudes and Restraint on the one hand, and BMI on the other, suggests an alternative explanation. The restraint subscale of the EDE-Q assesses not restricted eating per se but rather attempts at restricted eating: four of the five items include the words ‘tried’ , ‘trying’ , or ‘desire’ [13]. A low BMI, in contrast, is linked to actual restriction of food intake. This suggests the possibility that the Ascetic Attitudes component might be the outcome rather than the driver of success in attempts to restrict eating. This would be consistent with the concept of a common psychopathology that finds different forms of expression [20].

The failure of self-talk to predict OBE’s could be a reflection of the limited power of the study, with both components having a relationship approaching significance. However, while there were a number of participants with binge eating disorder (BED) in the main study, neither the literature nor the pilot study from which self-talk items were derived involved such individuals. If (BED) involves distinctive forms of self-talk, the inclusion of participants with this disorder in the study could have diluted the relationship between self-talk and OBE’s found in ED sufferers whose psychopathology is not limited to binge eating.

### Study limitations

In addition to the above, other limitations of the study should be acknowledged. The pilot study sample of six participants contained only one individual with a history of purging behaviours, with the remainder all having suffered from the restrictive type of AN. The failure (despite substantive efforts) to obtain a broader-based sample for the pilot, leaves open the possibility that important items of self-talk predominantly associated with bulimic behaviours were omitted from the study (in addition to possible items connected with (BED) mentioned above). The recruitment bias towards private sector clients, in both the pilot and main studies, should also be acknowledged.

A further issue concerns the lack of any formal ED diagnosis for 49% of participants assigned to the clinical sample. The positive predictive value reported by Mond et al. [14] for the criteria on which assignment was based was only 0.56, implying that approximately 20% of the clinical sample might not qualify for an ED diagnosis. Nevertheless, the fact that such substantial differences were found between the clinical and non-clinical samples on the CIA and on all subscales of the EDE-Q (Table 2), clearly indicates that the clinical sample represents individuals with extreme positions across the continua of ED psychopathology.

The study is also limited in providing no information as to how the relationship between self-talk and ED severity/psychopathology develops over time, both during the development of the disorder and as a result of successful treatment. The cross-sectional nature of the current study means that the extent to which self-talk remains a force driving the development/maintenance of the disorder, as Maisel et al. [1] would argue, as opposed to a mere symptom, remains open to argument.

### Future research

Further research is required to confirm the factor structure of ED self-talk to emerge from this exploratory study. Ideally, this research should be preceded by further small-scale pilot work amongst individuals with BN and (BED) to ensure that forms of self-talk specific to those disorders are not excluded. It should also be noted that the exploratory study intentionally involved a large number of self-talk categories, many of which were closely related (e.g., concerning alternative methods of compensating for food intake, and different forms of self-chastisement) to ensure potential differences were not lost. This initial study provides the basis for substantially reducing the number of items in future work.

Also of importance, as mentioned above, is to examine how the nature of self-talk and its relationship with ED severity/psychopathology develop over the course of the ED and in response to treatment. In this respect, it should be noted that Tierney and Fox [9] have suggested that the ED voice evolves over the development of the disorder, such that *Promises* become less prominent, and the abuser role components such as *Threats* and *Self-abuse* much more so. Confirming the extent of this change and, perhaps even more importantly, the process by which the sufferer’s own voice becomes locked (in factorial terms) with that of the ED, offers further considerable insight into the ED experience and as to how EDs can be more effectively treated. Additionally, one would wish to explore how ED self-talk changes over the course of recovery and in response to the differing therapeutic strategies employed to counter it.

### Clinical implications

This study serves to further clinical understanding of the self-talk experienced by ED sufferers and provides a basis for the more empathic approach to treatment advocated by Williams and Reid [8] and others. In particular, the fact that the component of self-talk that predicts overall ED severity is so characteristic of an abusive relationship suggests that too domineering an approach (argued by Maisel et al. [1] to be common practice in much ED treatment) risks adding to the client’s trauma and loss of sense of self. Rather, treatment needs to focus on empowering the ED sufferer to withstand the abuser that resides in her head. To this end, we note Tierney and Fox’s ([9], p. 252) exhortation to therapists to persevere with efforts to “infiltrate the relationship” between the sufferer and the eating disorder voice, and the advice of Vitousek, Watson, and Wilson [29] that therapists should seek to develop a collaborative relationship that employs a “judicious blend of empathy and firmness”.

Looking further at the structure of the crucial Abusive Relationship component suggests two areas of focus for clinical efforts. First, there may be value in loosening the connection between the negative appraisals of the abused self and the abusive voice of the ED so that the former can fulfil their potential as a force for change. Externalization offers one method by which this might be achieved. The second key point to emerge is that, in seeking to counter the impact of the ED voice, it is the ‘seducer’ and ‘abuser’ roles described by Maisel et al. [1] that would seem to merit primary attention. Fairburn et al. ([3], p. 119) suggests the patient be taught to recognise the “eating disorder mindset”. This data suggests that helping the patient identify specific types of the eating disorder mindset (e.g., seducer or abuser) may be helpful. Therapists might also be guided by the relationship between “the abuser” and purging and restrictive behaviour, and between ascetic attitudes and exercising and low weight, to more precisely target the type of mindset at work.

## Conclusion

The current study examined the relationship between ED self-talk, specific symptomatology, and overall severity. It identified a two factor component mode of self-talk in adult women; self-talk which reflects the ‘abusive relationship’ between the sufferer and the ED and ‘ascetic attitudes’ towards thinness. It supports the idea that self-talk plays an important role in ED maintenance and provides strong indications as to those aspects that most require addressing in therapy. It also brings us closer to understanding the experience of those who live with an ED.

## Competing interests

The authors received no financial support for the research, authorship, and/or publication of this article. The authors further declare that there are no potential conflicts of interest with respect to the research, authorship, and/or publication of this article.

## Authors’ contributions

NS conceived of and designed the study under the supervision of TLH. NS conducted the analysis of the literature and all pilot interviews, and designed the final questionnaire. NS also was responsible for publicising the study and generating the main sample. In this, he was substantially aided by CT. NS conducted the statistical analysis and wrote the first draft of the manuscript. TLH and CT have since contributed substantially to the final manuscript. All three authors have read and approved the final submission.
